# Awareness of Stem Cell Therapy for Diabetes Among Type II Diabetic Patients in Makkah: A Cross-Sectional Study

**DOI:** 10.7759/cureus.40981

**Published:** 2023-06-26

**Authors:** Lama S Almasoudi, Ghadi J Alqasimi, Rozan A AlHarbi, Rahaf S Alotaibi, Samah A Alharbi

**Affiliations:** 1 Faculty of Medicine, Umm Al-Qura University, Makkah, SAU; 2 Physiology Department, Faculty of Medicine, Umm Al-Qura University, Makkah, SAU

**Keywords:** type 2 diabetes mellitus, saudi arabia, treatment, level of awareness, stem cell therapy

## Abstract

Background

Diabetes mellitus is a chronic disease that affects millions of people worldwide. Several studies have suggested using stem cells for diabetes treatment. However, there is a lack of research assessing the population’s awareness of stem cells. This study aimed to evaluate the level of awareness regarding the use of stem cell therapy for type 2 diabetes mellitus (T2DM).

Methodology

This study was conducted from December 2021 to April 2022 through an online survey that was distributed electronically via social media platforms. T2DM patients or their care providers who lived in Makkah were included. Patients aged less than 18 years and those with mental disabilities were excluded.

Results

Of the 316 participants included in the study, 56% were males, 33% had an age range of 46-55 years, and 76% were married. T2DM patients and their caregivers had a moderate level of awareness about stem cell therapy, with caregivers having higher awareness than diabetic patients. A non-significant relationship was found between educational level, income, diabetes control, time of diagnosis, and patients’ awareness. However, regarding the decision of treatment, participants aged less than 35 years were highly likely to decide to undergo stem cell treatment compared to other age groups.

Conclusions

There is a moderate level of awareness about stem cell therapy as a treatment option for T2DM among T2DM patients and caregivers in Makkah. Hence, there is a need to raise awareness by using online and in-person well-organized education programs in Makkah.

## Introduction

Stem cells are unspecialized cells of the human body that can differentiate into any cell and have the ability to self-renewal. Stem cells exist both in embryos and adult cells. There are two major types of stem cells, namely, pluripotent stem cells which can differentiate into any cell type in the body, and multipotent stem cells which lack this trait and can only differentiate into a specific cell type. For example, multipotent cells derived from the gastrula’s mesoderm go through a differentiation step that restricts them to becoming only muscle and connective tissue; however, further differentiation results in increased specialization to only connective tissue until the cells can give rise to only cartilage or only bone [1].

To overcome the ethical and immunological issues associated with the use of human embryonic stem cells, scientists have developed a technique to reprogram/transform adult stem cells back to a pluripotent state [2].

Diabetes mellitus is a global illness that has become much more prevalent in recent years and is a leading cause of premature mortality [3]. Several studies have shown that using stem cell therapy can be a treatment option for type 2 diabetes mellites (T2DM) [4]. According to the World Health Organization, Saudi Arabia has the second-highest prevalence of diabetes among Middle East countries and the seventh-highest prevalence in the world [5].

It has been suggested that T2DM can be treated using stem cell therapy. Placenta-derived human mesenchymal stem cells have been shown to have the ability to improve the function of pancreatic islet cells, thus reducing blood glucose [2,6]. Furthermore, these cells have effective therapeutic effects in T2DM, including reduction of daily insulin requirement, improvement in the level of glycosylated hemoglobin (HbA1C), and decrease the blood glucose level [6]. Autologous human bone marrow-derived mesenchymal stem cells have been shown to have a short-term therapeutic impact in lowering HbA1C and fasting blood sugar levels in patients with T2DM who have been diagnosed for less than 10 years and are not obese [7]. Bone marrow-derived mesenchymal stem cells also help in the recovery of damaged pancreatic islet cells to a near-normal level [8].

In diet-induced diabetic mice, the use of human embryonic stem cell therapy showed no improvement in weight nor fasting blood glucose level; however, HbA1c levels were reduced and insulin sensitivity was mildly increased. A combination of anti-diabetic drugs and stem cell therapy may be effective in treating other types of diabetes [9]. Other studies using bone marrow mononuclear stem cell [10] and autologous bone marrow-derived stem cell [11] transplantation to treat T2DM patients have shown a significant reduction in fasting blood glucose and HbA1C levels, as well as a reduction in the number of medications they needed, with no significant side effects. Stem cell transplantation is considered to be a safer type of transplantation therapy for diabetes mellitus compared to whole-organ and islet transplantation [12].

There is a lack of studies on the assessment of the level of awareness and understanding of using stem cell therapy as an optional treatment for T2DM patients in Makkah. Therefore, we aimed to evaluate the level of awareness regarding the use of stem cell therapy for T2DM and compare different demographic backgrounds and how that affects the population’s knowledge.

This article was previously as a poster at the 1st Annual Saudi Medical Specialties Conference in Saudi German Hospitals, Makkah on March 18, 2023.

## Materials and methods

Study design

This community-based, cross-sectional, descriptive study was conducted in the region of Makkah, Kingdom of Saudi Arabia from December 2021 to April 2022. Study approval was obtained from the Biomedical Ethics Committee of Umm Al-Qura University (approval number: HAPO-02-K-012-2021-12-874).

Study population and sampling methodology

Adult residents of Makkah aged ≥18 years who were diagnosed with T2DM or their caregivers who agreed to participate were included in the study. Patients aged less than 18 years, those with mental disabilities, and those living outside Makkah were excluded.

Data collection

Data were collected through an original online questionnaire that was reviewed by a specialist. It was formulated in Arabic and English languages and completed using Google Forms. The questionnaire was distributed electronically via social media applications.

The questionnaire covered the following: participants’ sociodemographic data, including age in years, gender, nationality, residence, education, marital status, total perceived monthly family income, occupation, knowledge of stem cell therapy, would the participant use stem cell therapy in the future and the reason for his/her choice, and the source of information about stem cell therapy in T2DM. For patients, a question about the current status of diabetes mellitus control was added.

For the No answer, a score of 1 was given, and for the Yes answer, a score of 2 was given. The awareness levels were classified as follows: a low level of awareness with a score of less than 2.66, a moderate level of awareness with a score from 2.67 to 3.33, and a high level of awareness with a score from 3.34 to 4.

Data analysis

Data were analyzed using the SPSS® software for Mac, version 26 (IBM Corp., Armonk, NY, USA). For numerical variables, data were expressed as mean and standard deviation (mean ± SD). The t-test and analysis of variance test were used for data analysis. Spearman correlation was used to evaluate relationships involving ordinal variables. A p-value <0.05 was considered statistically significant.

## Results

A total of 316 participants fulfilled the inclusion criteria and were either diabetic patients or caregivers. Overall, 56% were males, 33% were aged from 46 to 55 years, and 76% were married. Further, 44% had a full-time job, 40% had a bachelor’s degree, and 34% had an income of less than 5,000 SR per month. Most patients 79% had children (Table 1). The mean body mass index of the participants was 28.7 ± 6.11 kg/m^2^, and the mean age of the youngest sibling was 34.5 ± 6.36 years.

**Table 1:** Description of the categorical factors of the participants.

Factor	Categories	Frequency	Percentage
Gender	Female	137	43%
	Male	179	56%
Age	Less than 35 years old	28	9%
	35–45 years old	36	11%
	46–55 years old	104	33%
	56–65 years old	86	27%
	66–75 years old	40	13%
	More than 75 years old	22	7%
Marital status	Single	26	8%
	Married	240	76%
	Divorced/Widow	50	16%
Education level	No degree	65	21%
	Secondary school	103	33%
	Bachelor’s degree	126	40%
	Master’s degree and above	22	7%
Income status	Less than 5,000 SR	108	34%
	5,000–10,000 SR	80	25%
	10,000–15,000 SR	63	20%
	More than 15,000 SR	65	21%
Employment status	Student/Unemployment	80	25%
	Part-time job	16	5%
	Full-time job	139	44%
	Retired	81	26%
Does the patient have children?	Yes	251	79%
	No	65	21%

Level of awareness about treatment options and stem cell therapy

As shown in Table 2, the mean awareness of all participants was 2.67, indicating that T2DM patients in Makkah had moderate awareness of stem cell therapy.

**Table 2:** Level of awareness.

One-sample statistics				
	N	Mean	Standard deviation	Standard error mean
Awareness of using stem cells	316	2.6741	0.79168	0.04454

There was a non-significant association between the time of diabetes mellitus diagnosis, education level, income status, and diabetes control and participants’ awareness level (p ≥ 0.05).

Differences in awareness between specific groups

The results showed that there was a significant difference in the awareness between diabetics and caregivers (p = 0.002), with caregivers having more awareness of stem cell therapy than diabetic patients.

Factors affecting the choices of treatment

The relationship between how seriously a complication impacted the choice of treatment was not significant (p = 0.357). When we evaluated the relationship between the patient’s preferences for stem cell therapy and their family history of morbidity, the result was non-significant (p = 0.113).

There was a difference between age groups and the decision of treatment of the patients which was statistically significant (p = 0.001). Table 3 illustrates which age groups were different from the others and shows that the age group (less than 35 years old) was mainly different.

**Table 3:** Age groups and choices.

Multiple comparisons					Multiple comparisons				
(I) Age groups	(J) Age groups	Mean difference (I-J)	Standard error	Significance	(I) Age groups	(J) Age groups	Mean difference (I-J)	Standard error	Significance
Less than 35 years old	35–45 years old	0.306	0.099	0.002	56–65 years old	Less than 35 years old	-0.360	0.086	0.000
	46–55 years old	0.260	0.084	0.002		35–45 years old	-0.055	0.078	0.483
	56–65 years old	0.360	0.086	0.000		46–55 years old	-0.101	0.057	0.080
	66–75 years old	0.375	0.097	0.000		66–75 years old	0.015	0.075	0.847
	More than 75 years	0.409	0.112	0.000		More than 75 years	0.049	0.094	0.606
35–45 years old	Less than 35 years old	-0.306	0.099	0.002	66–75 years old	Less than 35 years old	-0.375	0.097	0.000
	46–55 years old	-0.046	0.076	0.547		35–45 years old	-0.069	0.091	0.444
	56–65 years old	0.055	0.078	0.483		46–55 years old	-0.115	0.073	0.117
	66–75 years old	0.069	0.091	0.444		56–65 years old	-0.015	0.075	0.847
	More than 75 years	0.104	0.107	0.332		More than 75 years	0.034	0.105	0.745
46–55 years old	Less than 35 years old	-0.260	0.084	0.002	More than 75 years	Less than 35 years old	-0.409	0.112	0.000
	35–45 years old	0.046	0.076	0.547		35–45 years old	-0.104	0.107	0.332
	56–65 years old	0.101	0.057	0.080		46–55 years old	-0.149	0.092	0.107
	66–75 years old	0.115	0.073	0.117		56–65 years old	-0.049	0.094	0.606
	More than 75 years	0.149	0.092	0.107		66–75 years old	-0.034	0.105	0.745

Gender and the level of awareness were examined, and the results showed no significant correlation with a p-value of 0.290.

The impact of the age of the youngest sibling on their parents’ choices regarding therapeutic options was assessed, and the result showed that it was non-significant (p = 0.082).

## Discussion

In this study, we aimed to identify the level of awareness regarding stem cell therapy for diabetes among T2DM patients in Makkah, Saudi Arabia.

This study found that T2DM patients and their caregivers in Makkah have moderate awareness of stem cell therapy. An insufficient knowledge level was also reported in a previous Saudi study which aimed to assess the knowledge, attitude, and practice of doctors and medical students toward stem cells in diabetes mellitus management in Tabuk City. The study found that knowledge was poor in 21%, fair in 76.5%, and good in 2.5% of the participants [13].

In the Jouf region of Saudi Arabia, a study found a medium-to-high level of knowledge among the majority of students from healthcare sciences colleges, and a high attitude score was also noted toward stem cells [14]. The same poor knowledge was observed in a previous Malaysian study. This poor knowledge could be explained by a lack of exposure to stem cell therapy in our region [15].

A non-significant gender difference was found for awareness about stem cell treatment in this study. At the same time, this study found a non-significant relationship between the participants’ awareness and their income or educational level. Therefore, this study reveals that socioeconomic status does not have a significant effect on the awareness level regarding stem cell therapy for T2DM.

There is a lack of previous studies assessing the correlation between socioeconomic status and the awareness level of stem cell therapy in T2DM patients. The reason for this is that in Saudi Arabia, the Internet and social media are fairly accessible as they are affordable, available to everyone, and not dependent on monthly income. Moreover, the Ministry of Health organizes numerous free educational and awareness campaigns regarding any updated information. A study done in Saudi Arabia in Tabuk City found a non-significant relationship between knowledge level about stem cell treatment and participants’ demographics [13].

The current study also found that caregivers had a greater level of awareness compared to diabetic patients. A previous study found a similar result, where caregivers who lived with patients had a greater knowledge of diabetes in general [16].

This study found a non-significant association between the time of diabetes mellitus diagnosis, education level, income status, and diabetes control and participants’ awareness level. Due to a lack of studies assessing diabetic patients’ awareness of stem cells, it was difficult to find studies to compare our findings.

This study assessed if severe complications affect decisions regarding stem cell treatment in patients with T2DM. The study found a non-significant relationship between having diabetes-related comorbidities and the patient considering stem cell therapy. Although several treatments improve diabetes and help in delaying its complications, to date, there are no treatment options that completely cure diabetes [17]. This makes patients seek other treatments and encourages researchers to discover a curable medicine. Recently, extensive research on the use of mesenchymal stem cell (MSC) therapy to treat diabetes mellitus complications in pre-clinical animal studies has been conducted, and the majority of studies have shown effective outcomes for diabetic complications [18,19]. MSCs are anticipated to become effective therapeutic agents due to their potential for immunomodulatory ability, self-renewal, and differentiation [20].

Study limitations

A limitation of the present study was the use of a self-reporting questionnaire that could have a recall bias. In addition, the lack of research that addresses diabetic patients’ awareness of stem cells hindered the comparison between our study results with other studies. Finally, as the study was restricted to patients from Makkah, the results cannot be generalized, for which a country-wide sample is needed.

## Conclusions

This study demonstrates a moderate level of awareness about stem cell therapy among patients with T2DM and their caregivers in Makkah. The results showed that there is no significant association between the awareness about using stem cell therapy and the time of diagnosis, education level, income status, HbA1C level, and gender. On the other hand, the study found that caregivers were more aware of stem cell therapy.

The results revealed that severe complications, having children, dietary habits, and a family history of complications did not affect patients’ decision of using stem cell therapy. It was only affected by age as we found that patients aged less than 35 years had a higher possibility of deciding compared to the other age groups.

## Appendices

**Table 4:** English-language version of the questionnaire.

English-language version	
Consent form	The survey is part of a study conducted by a group of researchers and Dr. Samah Alharbi, PhD (Assistant professor, College of Medicine, Umm Al-Qura University) who is the principle investigator of this study. The target population for this study is the population of Makkah, Saudi Arabia. All information will be used only for the purpose of scientific research. Your participation in this questionnaire expresses your agreement to participate in this study, knowing that you can withdraw at any time by closing the questionnaire page
Questionnaire filler	Patient
	Caregiver
Demographic section:	
Patient’s age:	18–28
	29–39
	40–50
	51–61
	62–72
	73–83
	84 or older
Patient’s gender:	Male
	Female
Residency of the patient	Makkah
	Outside Makkah
Patient’s level of education:	High school or less
	Bachelor’s degree
	Master’s degree or higher
	No degree
Patient’s employment status:	Unemployed/Student
	Employed full-time
	Employed part-time
	Retired
Patient’s marital status:	Single
	Married
	Widowed
	Divorced
Does the patient have children?	Yes
	No
Patient’s weight:	------------
Patient’s height:	------------
Does the patient follow a diabetes-restricted diet?	Yes
	No
How often does the patient exercise?	Once or twice a week
	Once every month
	Every now and then
	Never
Income status of the patient/caregiver:	Less than 5,000 SR
	5,000–10,000 SR
	10,000–15,000 SR
	More than 15,000 SR
Diabetic status	
Has the patient been diagnosed with type 2 diabetes?	Yes
	No
When was the patient diagnosed with type 2 diabetes? (in years)	<1
	1–5
	6–10
	>10
The patient’s latest glycated hemoglobin (HbA1c) result:	------------
Does the patient have the following diabetes-related comorbidities? (You can choose more than one answer)	Diabetic nephropathy
	Diabetic eye complications
	Diabetic foot
	Diabetic cardiovascular complications
	Diabetic neuropathy
	None
	Others, ------------
Does the patient have a family history of diabetes?	Yes
	No
Does the patient have a family history of diabetes-related complications? (you can choose more than one answer)	Diabetic nephropathy
	Diabetic eye complications
	Diabetic foot
	Diabetic cardiovascular complications
	Diabetic neuropathy
	Death
	None
	Others, ------------
Knowledge about stem cell therapy for diabetes	
Has the patient heard about stem cell therapy?	Yes
	No
If yes, from where did he obtain this information?	Social media
	Relative/friend
	Your doctor/clinic advertisements
	Newspaper
	Television and radio
	Someone who got treated with stem cells
Has the patient thought about using stem cell therapy?	Yes
	No
If yes, why?	To be treated from diabetes forever
	To relieve his symptoms
	Fear of diabetes-related morbidity
	To stop using diabetes medication
	Doubt about the efficacy of his treatment
If no, why?	New treatment so fears of complications in the future
	Lack of information about the treatment
	cost
	Not available in Saudi Arabia
	This treatment was not recommended by his doctor
	Not approved by the Saudi Ministry of Health

**Table 5:** Arabic version of the questionnaire

النسخة العربية :	
هذا الاستبيان يهدف لقياس مدى معرفة مرضى السكري من النوع الثاني في مكة المكرمة عن خيارات التداوي التي تتضمن الخلايا الجذعية هذا الاستبيان جزء من دراسة يقوم بعملها مجموعة من الباحثين و دكتورة سماح الحربي ، دكتوراة (استاذ مساعد ، كلية الطب ، جامعة أم القرى ) وهي الباحثة الرئيسية في هذه الدراسة. الشريحة المستهدفة في هذه الدراسة هي كل سكان مكة المكرمة ، المملكة العربية السعودية. جميع المعلومات ستستخدم لغرض البحث العلمي فقط. مشاركتك في هذا الاستبيان تعني موافقتك على المشاركة في هذه الدراسة ،لذلك يمكنك الانسحاب في أي وقت عن طريق إغلاق صفحة الاستبيان.	الموافقة
المريض	من يقوم بملء الاستبيان
مقدم الرعاية	
المعلومات الشخصية والاجتماعية	
١٨-٢٨	عمر المريض
٢٩-٣٩	
٤٠-٥٠	
٥١-٦١	
٦٢-٧٢	
٧٣-٨٣	
٨٤ أو أكبر	
ذكر	جنس المريض
أنثى	
مكة	مكان إقامة المريض
خارج مكة	
ثانوية	مستوى تعليم المريض
بكالوريوس	
ماجستير أو اعلى من ذلك	
غير متعلم	
غير موظف/ طالب	حالة المريض الوظيفية
موظف بدوام كامل	
موظف بدوام جزئي	
متقاعد	
أعزب	حالة المريض الاجتماعية
متزوج/ة	
مطلق/ة	
أرمل/ة	
نعم	هل لدى المريض أطفال
لا	
------------	وزن المريض
------------	طول المريض
نعم	هل يتبع المريض حمية غذائية خاصة بحالته المرضية؟
لا	
مرة أو مرتان في الأسبوع	كم مرة يمارس المريض الرياضة ؟
مرة في الشهر	
نادرًا	
لا يمارس المريض الرياضة	
أقل من ٥٠٠٠ ريال سعودي	مستوى دخل المريض/ مستوى دخل مقدم الرعاية
٥٠٠٠-١٠٠٠٠ ريال سعودي	
١٠٠٠٠-١٥٠٠٠ريال سعودي	
أكثر من ١٥٠٠٠ ريال سعودي	
حالة السكري	
نعم	هل شُخص المريض بمرض السكري النوع الثاني؟
لا	
متى شُخص بمرض السكري ؟ ( بالسنوات)
	١-٥
	٦-١٠
	>١٠
_________	اخر قياس تراكمي
الفشل الكلوي	هل يعاني المريض من أحد مضاعفات من السكري ؟
اعتلال شبكية العين	
القدم السكرية	
أمراض القلب	
الاعتلال العصبي	
لا يوجد	
أخرى،ـــــــــــــــــــــــ	
نعم	هل لدى المريض تاريخ عائلي بمرض السكري ؟
لا	
الفشل الكلوي	هل لدى المريض تاريخ عائلي للإصابة بمضاعفات السكري ؟ (يمكنك اختيارأكثر من إجابة واحدة)
اعتلال شبكية العين	
القدم السكرية	
أمراض القلب	
الاعتلال العصبي	
وفاة	
لا يوجد	
أخرى،ـــــــــــــــــــــــ	
وعي مرضى السكري من النوع الثاني عن التداوي بالخلايا الجذعية	
نعم	هل سمع المريض عن استخدام الخلايا الجذعية في علاج مرض السكري ؟
لا	
وسائل التواصل الإجتماعي	اذا نعم، ما هو مصدر معلوماته ؟
أقارب / صديق	
طبيبك/إعلان دعائي من عيادة	
الصحف والجرائد	
التلفاز والراديو	
شخص عولج بالخلايا الجذعية	
نعم	هل فكر المريض في استخدام الخلايا الجذعية في علاج حالته المرضية؟
لا	
للتخلص من داء السكري	إذا كانت الإجابة نعم ،لماذا ؟
تخفيف حدة الأعراض	
التخوف من مضاعفات مرض السكري	
التوقف عن استخدام ادوية السكري	
الشك في فعالية الادوية	
دواء حديث بالتالي تخوف من الآثار الجانبية	
لا توجد معلومات كافية بخصوص هذا النوع من العلاج	إذا كانت الإجابة لا ،لماذا؟
التكلفة	
عدم تواجده في المملكة العربية السعودية	
لم يوصي به طبيب المريض	
لم تقره وزارة الصحة السعودية	
